# Ability of body mass index to predict abnormal waist circumference: receiving operating characteristics analysis

**DOI:** 10.1186/1758-5996-5-74

**Published:** 2013-11-19

**Authors:** Márcio Weissheimer Lauria, Lívia Maria Pinheiro Moreira, George Luiz Lins Machado-Coelho, Raimundo Marques do Nascimento Neto, Maria Marta Sarquis Soares, Adauto Versiani Ramos

**Affiliations:** 1 Internal Medicine, School of Medicine, Federal University of Minas Gerais, Belo Horizonte, MG, Brazil; 2 Endocrinology, Felicio Rocho Hospital, Belo Horizonte, MG, Brazil; 3 Medical Science, School of Medicine, Federal University of Ouro Preto, Ouro Preto, MG, Brazil

**Keywords:** Body mass index, Waist circumference, Obesity

## Abstract

**Background:**

Body mass index (BMI) and waist circumference (WC) are the most used anthropometric measures to identify obesity. While BMI is considered to be a simple and accurate estimate of general adiposity, WC is an alternative surrogate measure of visceral obesity. However, WC is subject to significant inter-examiner variation. The aim of the present study was to correlate BMI and WC measures in a group of Brazilian adults to determine the most accurate BMI values for predicting abnormal WC.

**Methods:**

BMI and WC were measured in 1184 volunteers (45.6 ± 17.3 yrs; 69% female) using standard procedures. Abnormal WC was defined as ≥88 cm in women and ≥102 cm in men using the traditional criteria, and ≥80 cm in women and ≥90 cm in men using the new criteria. Statistical analysis involved the calculation of Pearson’s correlation coefficients and receiver operating characteristic (ROC) curves.

**Results:**

BMI was strongly correlated with WC (women: *r* = 0.87, *p* < 0.0001, area under ROC curve = 0.93 ± 0.1; men: *r* = 0.89, *p* < 0.0001, area under ROC curve = 0.94 ± 0.01). The most accurate BMI cutoff point for abnormal WC was 27.1 kg/m^2^ for men and 26.8 kg/m^2^ for women using the traditional WC criteria, and 24.7 kg/m^2^ for men and 24.9 kg/m^2^ for women using the new WC criteria.

**Conclusion:**

Based on the strong correlation found with WC, BMI can be used as the primary anthropometric measure to estimate adiposity, since both obese and most overweight subjects will have abnormal WC.

## Background

Obesity is the fastest-growing chronic disease in both children and adults, and is becoming a global epidemic. In 2008, an estimated 1.5 billion adults worldwide were considered to be overweight or obese [1]. This public health concern is the cause of a number of comorbidities, being associated with high health care costs. Obesity is a risk factor for a variety of diseases, such as type 2 diabetes mellitus, cardiovascular disease, hypertension and cancer [2-4].

Overweight in adults is defined as a body mass index (BMI = weight in kilograms/height in meters squared) of 25.0 to 29.9 kg/m^2^, while obesity is defined as a BMI ≥ 30.0 kg/m^2^[5]. BMI was first described by Adolphus Quetelet in the mid- nineteenth century, based on the observation that body weight is proportional to height squared in adults with normal body frames [6]. BMI is widely employed as an anthropometric estimate of general adiposity.

In recognition that visceral fat accumulation increases the risk of metabolic disease, waist circumference (WC) has been used as an alternative surrogate measure of obesity. Abnormal WC is traditionally defined as ≥88 cm in women and ≥102 cm in men, based on cutoff points advocated by Lean et al. in 1995 [7]. Recently, however, new cutoff points have been proposed for abnormal WC taking into consideration ethnic aspects. In South Americans, abnormal WC is defined as ≥80 cm in women and ≥90 cm in men. These values are based on the definition of metabolic syndrome by the International Diabetes Federation (IDF) [8].

However, while weight and height measurements are relatively simple and accurate, WC is subject to significant inter-examiner variation, which leads to concerns regarding its reliability. Furthermore, BMI has been described as a better predictor of cardiovascular events than WC in some studies [9-11].

The aim of the present study was to correlate BMI and WC in a group of Brazilian adults to determine the most accurate BMI values that best predict abnormal WC in men and women.

## Methods

A total of 1184 subjects were consecutively evaluated and included in the present study. The volunteers spontaneously presented for a medical appointment at a general endocrine clinic at the Felício Rocho Hospital, Brazil, between March 2011 and February 2012. The exclusion criteria were the following: age < 18 yrs, history of abdominoplasty, history of solid organ transplant and chronic kidney disease.

All the data were evaluated by physicians trained in measuring weight, height and WC using standard techniques. BMI was obtained from height and weight measurements with the subjects barefoot and wearing light clothing. A scale with accurate weighing up to 150 kg was used for weight determination. Height was measured to the nearest 1 cm using a stadiometer. For this measurement, the participants were positioned with heels, buttocks, shoulder blades and back of the head in contact with the backboard of the stadiometer and the top of the head in the Frankfort horizontal plane. BMI was calculated as weight in kilograms divided by height in meters squared (kg/m^2^).

WC was measured with the subject in the standing position, at the end of exhalation, using a non-elastic measuring tape placed horizontally at the midpoint between the lowest rib and top of the iliac crest, as defined by the World Health Organization. Two different cutoff points were used for establishing abnormal WC: 1) ≥88 cm in women and ≥102 cm in men (traditional criteria); and 2) ≥ 80 cm in women and ≥90 cm in men (new criteria). The subjects were allocated into two groups (normal and abnormal) based on these two cutoff points.

To estimate the most accurate BMI cutoff point for discriminating normal from abnormal WC, receiving operating characteristic (ROC) curves were constructed and separated by gender using both WC classification criteria. The point closest to the maximum sensitivity and specificity point was selected as the cutoff.

Correlation analyses were performed between the BMI and WC using Pearson’s correlation coefficient (r) and area under ROC curves with 95% confidence interval {AUC (95% CI)}. An *r* value >0.7 indicated a strong correlation; *r* = 0.4 to 0.7 indicated a moderate correlation, and *r* <0.4 denoted a weak correlation. The Prism 4.0 software (Graph Pad, San Diego, CA, USA) was used for all analyses and a *p* value <0.05 was deemed statistically significant.

## Results and discussion

Mean patient age was 45.6 ± 17.3 yrs. Women accounted for 69% of the sample. Mean BMI was 25.4 ± 4.8 kg/m^2^ for men and 27.1 ± 5.9 kg/m^2^ for women. Mean WC was 89.9 ± 14.4 cm for men and 88.9 ± 15.1 cm for women.

**Figure 1:**
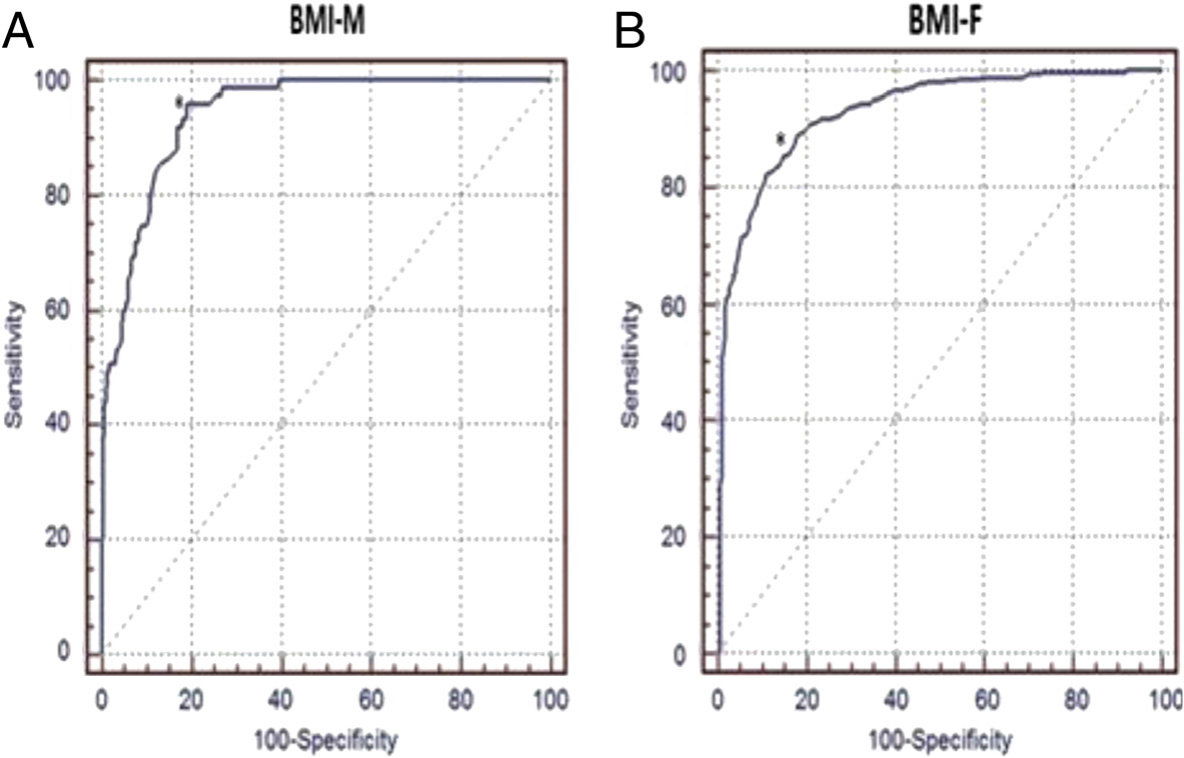
**ROC curve for prediction of central obesity (WC = 102 cm in men and 88 cm in women). (A)** for men: cut-off value = 27.1 kg/m^2^ {sensitivity: 96%; specificity: 81%, AUC (95% CI) = 0.94 (0.91-0.96)} **(B)** for women: cut-off value = 26.8 kg/m^2^ {sensitivity: 82%; specificity: 89%, AUC (95% CI) = 0.93 (0.91-0.95)} AUC (95% CI) = area under ROC curves with 95% confidence interval; BMI-F = Body mass index in men, BMI-M = Body mass index in men.

**Figure 2:**
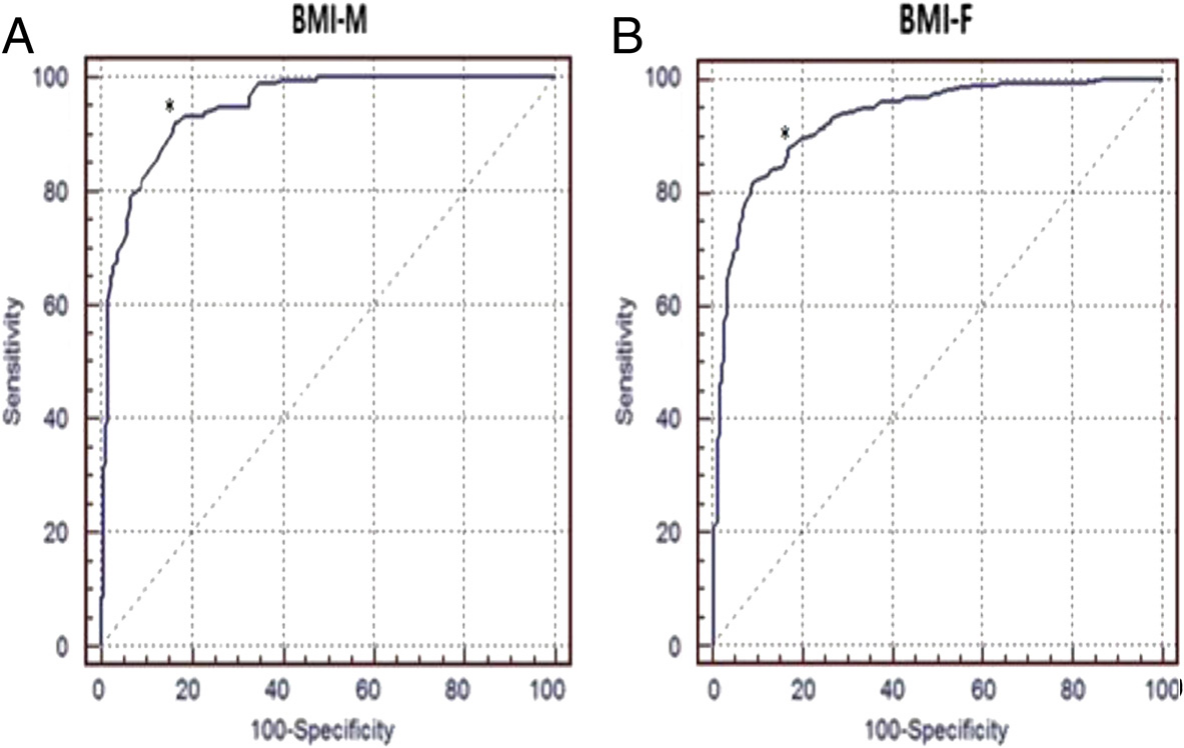
**ROC curve for prediction of central obesity (WC = 90 cm in men and 80 cm in women). (A)** for men: cut-off value = 24.7 kg/m^2^ for men {sensitivity: 92%; specificity: 84%, AUC (95% CI) = 0.95 (0.92-0.97)}. **(B)** for women: cut-off value = 24.9 kg/m2 for women {sensitivity: 82%; specificity: 91%, AUC (95% CI) = 0.93 (0.91-0.94)}. AUC (95% CI) = area under ROC curves with 95% confidence interval; BMI-F = Body mass index in men, BMI-M = Body mass index in men.

Pearson’s correlation coefficient between BMI and WC was 0.89 for men (*p* < 0.0001) and 0.87 for women (*p* < 0.0001). The AUC (95% CI) between BMI and WC using the traditional classification was 0.94 (0.91-0.96) for men and 0.93 (0.91-0.95) for women (Figure 1). The AUC (95% CI) between BMI and WC using the new criteria was 0.95 (0.92-0.97) for men and 0.93 (0.91-0.94) for women (Figure 2).

The most accurate BMI cutoff point for abnormal WC was 27.1 kg/m^2^ for men (sensitivity: 96%; specificity: 81%) and 26.8 kg/m^2^ for women (sensitivity: 82%; specificity: 89%) using the traditional classification (Figure 1). Using the new criteria, the most accurate BMI cutoff point for abnormal WC was 24.7 kg/m^2^ for men (sensitivity: 92%; specificity: 84%) and 24.9 kg/m^2^ for women (sensitivity: 82%; specificity: 91%) (Figure 2).

The present cross-sectional study with >1000 subjects demonstrated a strong correlation between BMI and WC in both genders. This study involved patients at ages ranging from 18 to 90 yrs. Moreover, no distinctions were made regarding ethnicity. In fact, considering the ethnic mix of the Brazilian population, the present findings may also apply to other populations. Strong correlation between BMI and WC (80-85%) has also been described in large cohorts [9,10]. Therefore, BMI provides a good estimation of WC in the majority of patients and can reasonably replace it, which is a very convenient feature in clinical practice.

BMI is the most widely employed anthropometric measure, due in part to its convenience, safety and minimal cost. Several studies have related BMI to mortality and morbidity rates, thereby demonstrating that subjects with normal BMI or slightly overweight are in the lower risk group [12]. A recent meta-analysis found that obesity class 2 and 3 (BMI = 35–39.9 and ≥ 40 kg/m^2^, respectively) were significantly associated with increased overall mortality, while class 1 obesity (BMI = 30–34.9 kg/m^2^) was not associated with higher mortality rates and overweight was actually associated with significantly lower overall mortality rates [13].

On the other hand, using BMI alone for obesity assessment could be a limitation because it reflects both fat and lean mass and does not discriminate fat distribution [14]. Okorududu et al. conducted a systematic review of studies evaluating the performance of BMI in the detection of body adiposity and found that cutoff points for the diagnosis of obesity have high specificity but low sensitivity regarding the identification of adiposity, as half of the individuals with a high percentage of fat were not identified using this measure [15]. Moreover, BMI is particularly inaccurate in subjects with elevated lean body mass, such as athletes, and cannot be generalized among different ethnic groups. This measure is also criticized because it makes no difference between men and women [16].

WC, in turn, provides an estimation of abdominal fat and has been related to insulin resistance and cardiovascular risk. It is the cornerstone of the IDF definition of metabolic syndrome [8]. However, WC measurement is frequently unreliable, since it varies depending on the precise site at which the measurement is performed [17,18]. A study published in 2008 determined excellent inter-observer reliability for weight, height and derived BMI (*r* >0.99), but unsatisfactory reliability for WC (*r* =0.92). Only 1% of volunteers were misclassified as overweight or obese based on the BMI, whereas the use of WC led to misclassification in 6% of cases [19]. Moreover, WC is equal or inferior to BMI in predicting mortality or cardiovascular disease, according to recent data from large cohorts [9-11]. These results challenge current recommendations on obesity-related cardiovascular risk management based on WC and underscore the need for further research to improve the reliability of anthropometric measurements by physicians.

In 2006, IDF recommended modifications in the definition of abnormal WC, taking into account ethnic aspects [8]. In the present study, the impact of these modifications in the relation between WC and BMI was demonstrated. Using the traditional criteria, BMI above 27 kg/m^2^ was the most accurate cutoff point for the prediction of abnormal WC in both genders. Interestingly, the use of the new and more stringent criteria determined that all overweight/obese subjects had abnormal WC and the most accurate cutoff point was approximately 25 kg/m^2^ in both men and women. This represents a controversial issue since overweight has been associated with significantly lower mortality overall relative to the normal weight category [13].

## Conclusions

Based on the present findings of strong correlation with WC, BMI can reasonably be used as the first anthropometric measure to estimate adiposity since obese and the majority overweight subjects will have abnormal WC. For these patients, WC measure can be waived. For patients with a lower BMI, WC remains informative and has yet to be determined.

## Abbreviations

AUC (95% CI): Area under ROC Curves with 95% confidence interval; BMI: Body mass index; IDF: International diabetes federation; R: Pearson’s correlation coefficient; ROC: Receiving operating characteristics; WC: Waist circumference.

## Competing interests

The authors have no conflicts of interest and this study was not supported by any pharmaceutical company.

## Authors’ contributions

MWL 1) have made substantial contributions to conception and design, or acquisition of data, or analysis and interpretation of data; 2) have been involved in drafting the manuscript or revising it critically for important intellectual content; LMPM 1) have made substantial contributions to conception and design, or acquisition of data, or analysis and interpretation of data; 2) have been involved in drafting the manuscript or revising it critically for important intellectual content; GLLM-C 1) have made substantial contributions to conception and design, or acquisition of data, or analysis and interpretation of data; RMdoNN 1) have made substantial contributions to conception and design, or acquisition of data, or analysis and interpretation of data; MMSS Have given final approval of the version to be published. AVR Have given final approval of the version to be published. All authors read and approved the final manuscript.
